# A rare cause of lower gastrointestinal bleeding treated with robotic colorectal surgery

**DOI:** 10.1186/s40792-021-01207-6

**Published:** 2021-05-20

**Authors:** Robin Osofsky, Cyril Kamya, Hamza Hanif, Victor Phuoc

**Affiliations:** 1 Department of Surgery, UNM Hospital - 2ACC, University of New Mexico Hospital School of Medicine, Albuquerque, NM 87131 USA; 2 Shifa College of Medicine, Sector H-8/4, Islamabad, 44000 Federal Capital Pakistan

**Keywords:** Metastatic melanoma, Sigmoidectomy, Sigmoid colectomy, Robotic surgery, Da Vinci

## Abstract

**Background:**

Metastatic melanoma to the colon is rarely diagnosed with an incidence of only 0.3% and more than 95% of cases identified post-mortem. Survival for patients with metastatic melanoma to the colon is poor, with 5-year survival rates of 26.5%. Nonetheless, surgical resection of the colonic metastatic melanoma lesions is recommended as it is associated with improved survival. Additionally, surgical resection is also indicated for palliative reasons, as symptom resolution is achieved in 90% of such patients. Use of the surgical robot has increased dramatically in the past decades, especially in the field of colorectal surgery. Furthermore, recent studies have demonstrated comparable outcomes between patients undergoing either laparoscopic or robotic-assisted colorectal surgery for cancer. Here, we describe the first case, to the authors knowledge, of a robot-assisted sigmoid colectomy for metastatic melanoma.

**Case presentation:**

A 72-year-old male with a history of metastatic melanoma diagnosed in 2015 with a favorable response to immunotherapy presented to the emergency department with symptomatic lower gastrointestinal bleeding (LGIB). Endoscopy demonstrated a friable melanotic lesion of the sigmoid colon with biopsy demonstrating histopathologic evidence of metastatic melanoma. After further evaluation, the patient consented for an elective robot-assisted segmental colectomy for palliative intent. Diagnostic laparoscopy identified no evidence of further intra-abdominal metastatic disease. After identifying the metastatic lesion in the sigmoid colon, the mesentery of involved segment of sigmoid colon adjacent to the lesion was divided using the bipolar electrosurgical vessel sealer device. The colon was divided both proximal and distal to the lesion using a robotic stapler and a tension-free colo-colonic anastomosis was created intracorporeally. Postoperatively, the patient had an unremarkable course and was discharged home on post-operative day 3. On follow-up, the patient was doing well with resolution of preoperative LGIB.

**Conclusion:**

This case highlights a rare presentation of metastatic melanoma to the colon in a patient presenting with LGIB. Furthermore, this case demonstrates the feasibility of the minimally invasive robotic-assisted approach for an uncommon pathology.

## Background

Metastatic melanoma is the most lethal type of skin cancer and has rapid systemic dissemination [1], with a 5-year survival rate less than 15% in patients with metastatic disease [2]. Furthermore, colonic metastasis from primary cutaneous melanoma is extremely rare, with a reported incidence of only 0.3% in such patients [3]. However, over 95% of gastrointestinal (GI) metastases from primary cutaneous melanoma are discovered post-mortem [4]. In addition, GI metastases are identified on autopsy in half of all patients with disseminated melanoma [5]

The mean time from time of diagnosis of primary melanoma to the diagnosis of colonic metastasis ranges from 31.7 to 89.6 months [3, 6]. Patients with colonic metastasis from a primary cutaneous melanoma most commonly present with lower gastrointestinal bleeding (LGIB), abdominal pain, or obstructive symptoms [5]. Hence, a thorough investigation for GI metastasis is recommended in any patient with such symptoms, as surgical resection can offer both palliative and survival benefits [7, 8].

The use of the surgical robot has become increasingly widespread, particularly in the field of colorectal surgery [9]. Robotic-assisted surgery offers a multitude of advantages compared to traditional laparoscopy including three-dimensional visualization, increased degrees of freedom, motion scaling, ergonomic positioning, shortened learning curve, and elimination of the fulcrum effect [9–11]. Such features have allowed for a broader operative field in narrow places, such as the pelvis, which is of paramount importance in colorectal surgery. We describe the utility of the surgical robot in the management of primary cutaneous metastatic melanoma of the sigmoid colon.

## Case presentation

We present a 72-year-old male with a past history significant for metastatic melanoma, hypertension, hyperlipidemia, type-2 diabetes mellitus, gout, chronic pancreatitis, adrenal insufficiency secondary to immunotherapy, and BMI of 30. The patient was incidentally diagnosed with metastatic melanoma in 2015 after presenting to the emergency department with shortness of breath. Subsequent imaging identified pancreas, brain, and pulmonary lesions, which were later confirmed to be metastatic melanoma after undergoing thoracoscopic wedge resection. Since time of diagnosis, the patient’s oncologic treatment has consisted of chemotherapy, stereotactic radiotherapy, and immunotherapy with pembrolizumab to which he had shown good response.

**Fig. 1:**
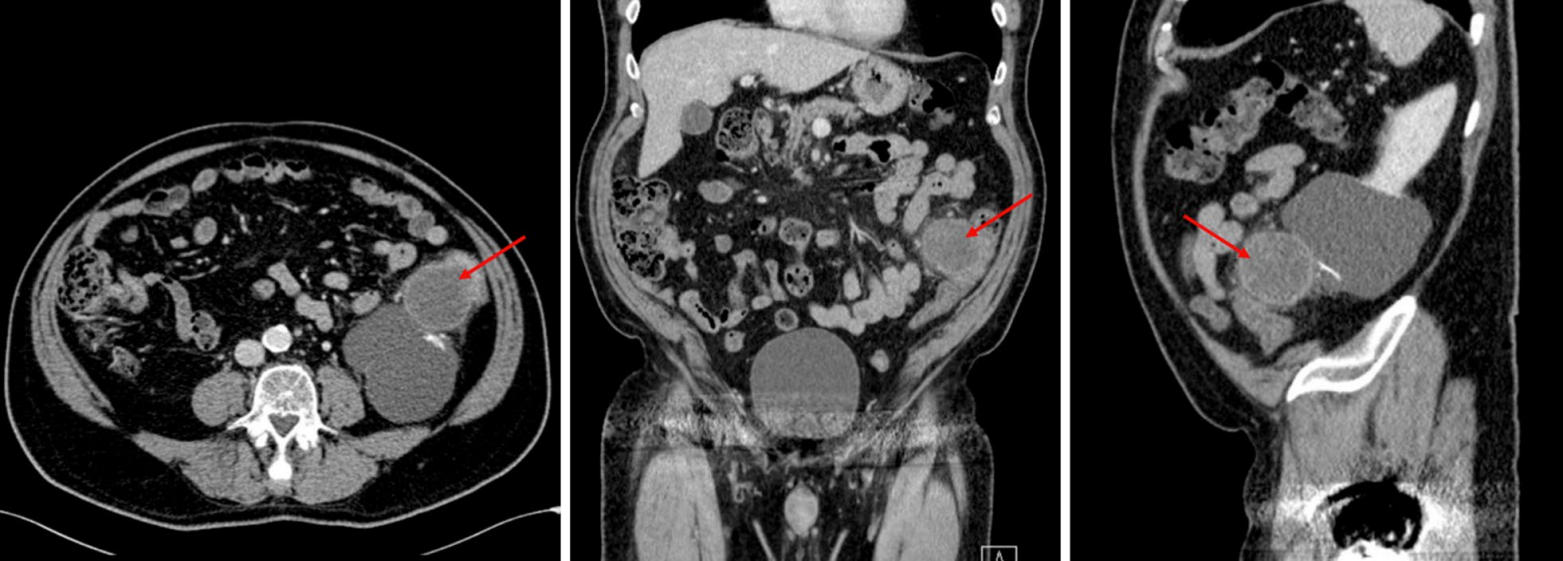
Computed tomography (CT) scan of abdomen and pelvis. Axial, coronal, and sagittal views are shown (left to right), respectively. The figure demonstrates the metastatic lesion in the in left lower quadrant of the abdomen (arrow)

In late 2018, the patient presented to the emergency department with symptomatic LGIB. A subsequent computed tomography (CT) scan of the abdomen and pelvis demonstrated a 6.3-cm lesion of the large intestine in the left lower quadrant, shown in Fig. 1. The patient subsequently underwent a colonoscopy which demonstrated a friable mass invading the lumen of the sigmoid at 40 cm from rectum, with pathology consistent with primary cutaneous metastatic melanoma. After discussion at the multidisciplinary surgical oncology conference, the recommendation that the patient to undergo a robotic-assisted sigmoid colectomy with primary anastomosis.

**Fig. 2:**
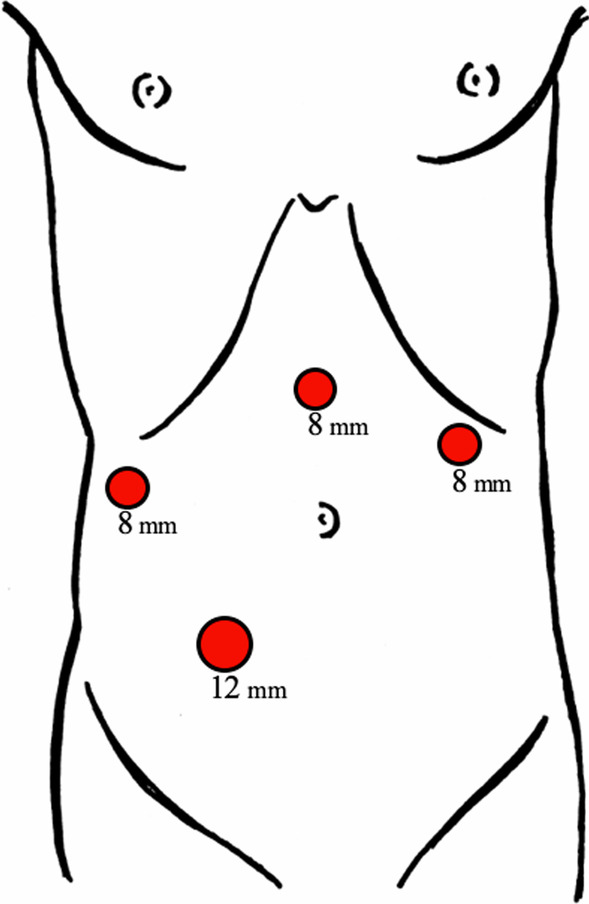
Trocar site placement: Palmer’s point (8 mm), upper midline (8 mm), right flank (8 mm), and right lower quadrant (12 mm)

Under general anesthesia, the patient was placed in supine position, arms tucked. The abdominal cavity was accessed via optical trocar entry technique at Palmer’s point with an 8-mm trocar. Additional 8-mm trocars were placed in upper midline and right flank. The right lower quadrant was chosen as the extraction site, via a 5-cm muscle-splitting incision through which a wound protector was placed. A gelport was positioned over this wound protector, and a 12-mm trocar was inserted. Abdominal trocar sites are depicted in Fig. 2. The surgical robot *(DaVinci, Xi*, Intuitive Surgical Inc., Sunnyvale, CA) was docked, and robotic instruments were inserted.

Robotic diagnostic laparoscopy showed no evidence of peritoneal or hepatic metastatic disease. The descending colon and sigmoid colon were mobilized by dividing the lateral retroperitoneal attachments using monopolar electric cautery. The metastatic lesion was apparent on the mesenteric aspect of the proximal sigmoid colon. The mesentery of involved segment of sigmoid colon adjacent to the lesion was divided using the bipolar electrosurgical vessel sealer device, while ensuring adequate hemostasis. Notably, as the procedure was for palliative rather than curative intent, a regional lymphadenectomy along the inferior mesenteric artery was not performed. The colon was divided both proximal and distal to the lesion using a robotic 45-mm stapler with 3.5-mm staples. To achieve a tension-free colo-colonic anastomosis, mobilization of the splenic flexure was required, and dissection was performed in a medial to lateral fashion.

**Fig. 3:**
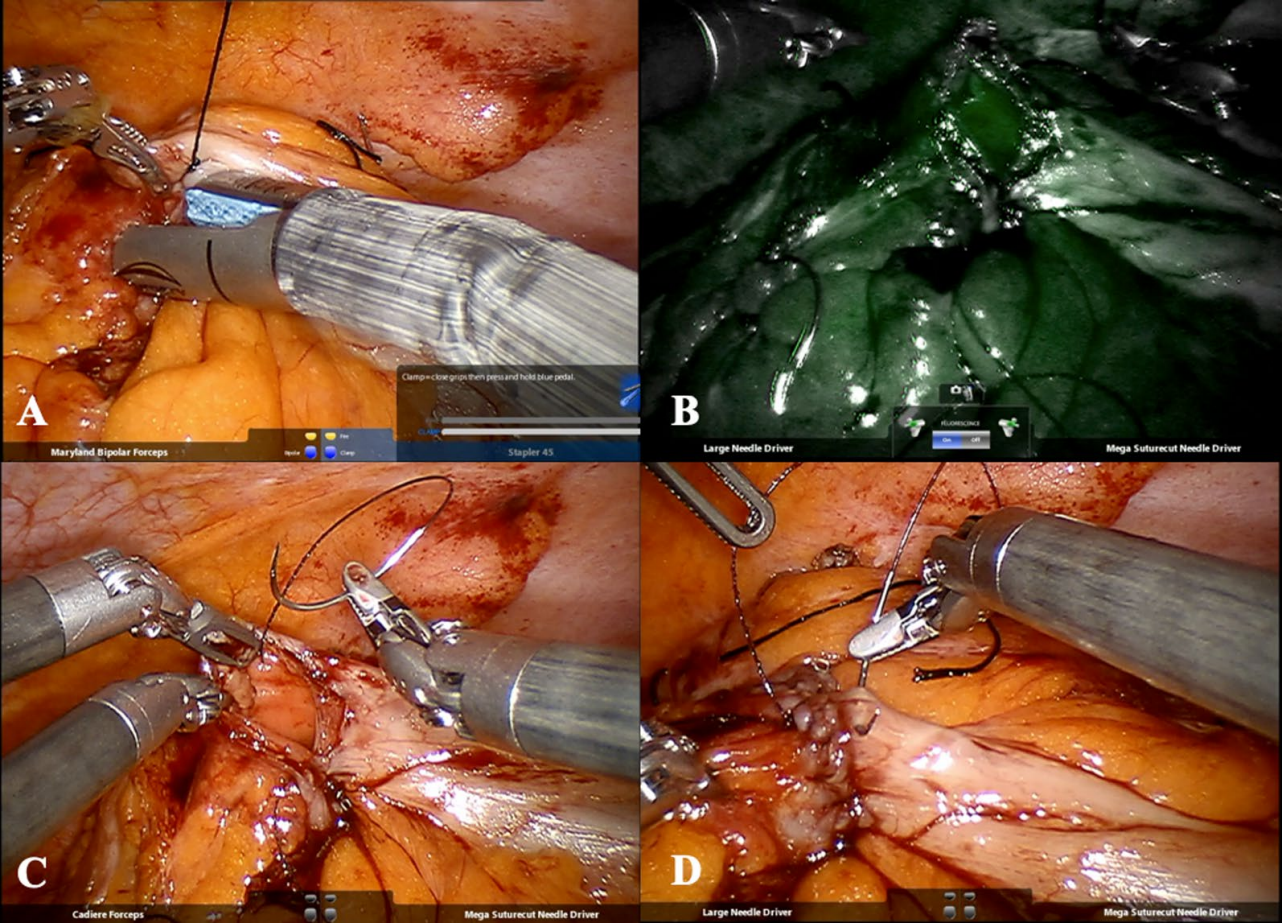
Creation of robotic-assisted intracorporeal colo-colonic anastomosis. **a** Creation of common channel utilizing the robotic 45-mm stapler with 3.5 mm. **b** Intraoperative indocyanine green fluorescence angiography demonstrating adequate anastomotic perfusion. **c** First-layer closure of common enterotomy in running fashion. **d** Second-layer close of common enterotomy in Lembert fashion

The anastomosis was created by first orienting the proximal resection and distal resection lines of the descending and sigmoid colon, respectively, in an isoperistaltic fashion utilizing silk stay sutures. Enterotomies were created using monopolar cautery on the proximal and distal limb. A common channel was created using the robotic 45-mm stapler with 3.5-mm staples to create the isoperistaltic side-to-side anastomosis, shown in Fig. 3a. Adequate anastomotic perfusion was ensured by via intraoperative indocyanine green fluorescence angiography, shown in Fig. 3b. The resulting common enterotomy was closed with an absorbable barbed suture in two layers; the first layer in a running fashion and the second layer imbricated in a Lembert fashion, shown in Fig. 3c and d, respectively. The specimen was then delivered through the right lower quadrant extraction site utilizing a wound protector site, and the abdomen was closed.

**Fig. 4:**
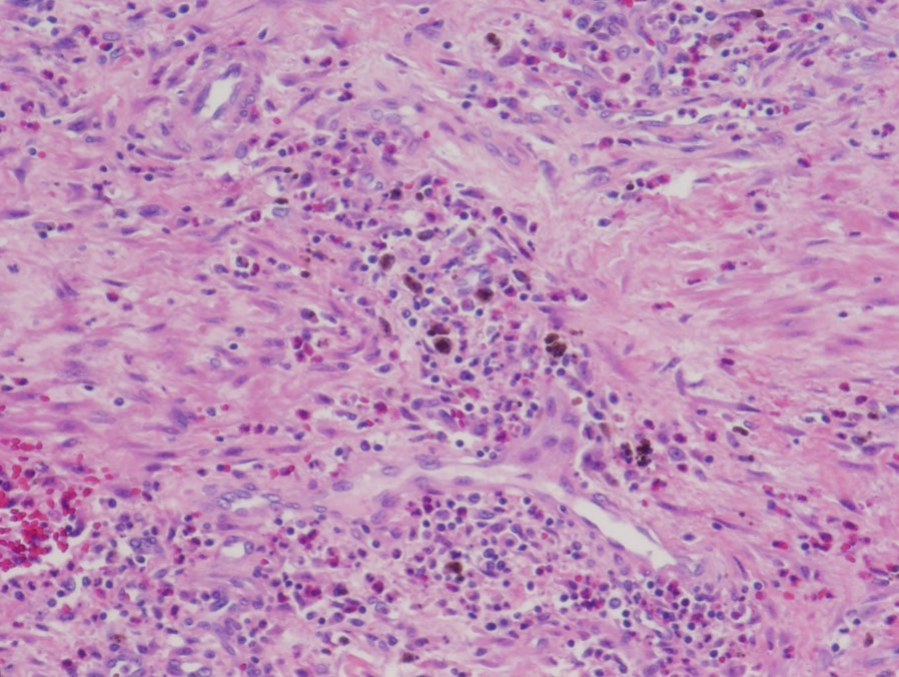
Hematoxylin and eosin-stained slide demonstrating metastatic melanoma within the wall of the sigmoid colon

Total operative time was 238 min, with an estimated blood loss of only 50 mL. Postoperatively, the patient had adequate pain control and was tolerating clear-liquid diet by the post-operative day zero. He was advanced to regular diet over the next two days as tolerated. By post-operative day three, the patient was ambulating well, his pain was well-controlled on oral pain medication, he had return of normal bowel function, and was subsequently discharged home. The pathology demonstrated metastatic melanoma of the colon, with ulceration and necrosis. A representative image of the specimen is depicted in Fig. 4. Zero out of 12 lymph nodes was found to be positive for disease and the resection margins were negative. On 1-month follow-up, the patient was without pain, tolerating a regular diet, and with resolution of his preoperative hematochezia.

## Discussion

The most common sites for melanoma metastasis are the lung, brain, liver, bone, and intestine [2, 12]. Melanoma has a described metastatic affinity to the small intestines, with gastric and large intestine being less common [7]. Large autopsy studies have demonstrated GI metastases in up to 60% of patients with metastatic melanoma [6, 13]. However, the small intestine harbors the greatest proportion of metastatic melanoma of the GI tract at 91% [5]. Furthermore, metastatic melanoma to the small intestine is associated with a 5-year survival rate of < 5% [14].

Metastatic melanoma to the large intestine is far less common, occurring in only 0.3% of patients with primary melanoma, with sigmoid colon involvement in 18.3% of cases [3, 6]. Survival for patients with metastatic melanoma to the colon is poor, with median survival of 31.7 months and 5-year survival rates of 26.5% [6]. Nonetheless, surgical resection of the colonic metastatic melanoma lesions is recommended as it is associated with improved survival compared to those treated without resection [3, 5, 7, 8, 14]. Surgical resection is also indicated for palliative reasons, as symptom resolution is achieved in 90% of the patients with colonic metastases following palliative resection [5].

Negative prognostic factors for survival of patients with metastatic melanoma to the colon include node positivity, extracolonic metastases, and colonic perforation or obstruction [3, 8]. In our case presentation, the patient’s metastatic disease involved lungs, brain, and pancreas, hence the goal of the operation was for palliative rather than curative intent. Additionally, the patient previously had a favorable response to pulmonary wedge resection, chemotherapy, and immunotherapy. Hence, a decision was made to perform a segmental colonic resection, as an R0 resection of the tumor could provide symptom relief and improve overall survival [5, 15]. We opted to approach the resection in a minimally invasive manner utilizing the surgical robot.

Minimally invasive approaches have become routine in the practice of colorectal surgery. A landmark study investigating laparoscopic versus open colectomy for cancer found that, disease-free 5-year, overall 5-year survival, overall recurrence rates, and complications complication rates were comparable between laparoscopic and open groups [16]. Although laparoscopy was associated with longer operative time, patients in the laparoscopic group had shorter hospital stays, decreased analgesic use, and more rapid return of bowel function [16]. In our case, a minimally invasive approach allowed for diagnostic laparoscopy in order to rule out disseminated intra-abdominal metastasis.

In recent decades, the use of robotic surgery has grown exponentially, particularly in the field of colorectal surgery [10]. Following 2007, the number of robotic-assisted procedures increased worldwide from 80,000 to over 200,000 [17]. In our case, the added degrees of freedom associated with the surgical robot [9–11], allowed for improved suturing and facilitated creation of an intracorporeal anastomosis.

Several comparative studies have been conducted between the surgical robot and conventional laparoscopy. A commonly described advantage of the surgical robot in colorectal surgery is a decreased rate of conversion to open compared to conventional laparoscopy [9, 18, 19]. Additionally, in patients undergoing laparoscopic or robotic-assisted colorectal surgery for cancer, there were no differences in length of hospital stay, return of bowel function, rate of complications, or oncologic outcomes [18, 19].

This case report provides an example of successful utilization of the surgical robot in rare patient pathology, thus providing evidence for more routine use of this technology. Additionally, as robotic-assisted surgery has a shorter learning curve than traditional laparoscopy [9, 20], the barrier to entry for adopting a new surgical skillset is decreased. Thus, further adoption of robotic-assisted surgery will allow for more surgeons to perform minimally invasive procedures.

## Conclusions

In conclusion, this case highlights a rare presentation of metastatic melanoma to the colon in a patient presenting with LGIB. Additionally, it demonstrates the feasibility of the minimally invasive robotic-assisted approach for an uncommon pathology requiring segmental colectomy, provided careful selection and adequate surgeon experience.

## Data Availability

The datasets obtained during this study are available from the corresponding author on reasonable request.

## Abbreviations

GI: Gastrointestinal
LGIB: Lower gastrointestinal bleeding
CT: Computed tomography
